# The NHGRI-EBI GWAS Catalog of published genome-wide association studies, targeted arrays and summary statistics 2019

**DOI:** 10.1093/nar/gky1120

**Published:** 2018-11-16

**Authors:** Annalisa Buniello, Jacqueline A L MacArthur, Maria Cerezo, Laura W Harris, James Hayhurst, Cinzia Malangone, Aoife McMahon, Joannella Morales, Edward Mountjoy, Elliot Sollis, Daniel Suveges, Olga Vrousgou, Patricia L Whetzel, Ridwan Amode, Jose A Guillen, Harpreet S Riat, Stephen J Trevanion, Peggy Hall, Heather Junkins, Paul Flicek, Tony Burdett, Lucia A Hindorff, Fiona Cunningham, Helen Parkinson

**Affiliations:** 1 European Molecular Biology Laboratory, European Bioinformatics Institute, Wellcome Genome Campus, Hinxton, Cambridge CB10 1SD, UK; 2 Open Targets, Wellcome Genome Campus, Hinxton, Cambridge CB10 1SD, UK; 3 Wellcome Sanger Institute, Wellcome Genome Campus, Hinxton, Cambridge CB10 1SA, UK; 4 JDRF/Wellcome Trust Diabetes and Inflammation Laboratory, Wellcome Centre for Human Genetics, University of Oxford, NIHR Oxford Biomedical Research Centre, Nuffield Department of Medicine, Oxford, UK; 5 Division of Genomic Medicine, National Human Genome Research Institute, National Institutes of Health, Bethesda, MD 20892, USA

## Abstract

The GWAS Catalog delivers a high-quality curated collection of all published genome-wide association studies enabling investigations to identify causal variants, understand disease mechanisms, and establish targets for novel therapies. The scope of the Catalog has also expanded to targeted and exome arrays with 1000 new associations added for these technologies. As of September 2018, the Catalog contains 5687 GWAS comprising 71673 variant-trait associations from 3567 publications. New content includes 284 full *P*-value summary statistics datasets for genome-wide and new targeted array studies, representing 6 × 10^9^ individual variant-trait statistics. In the last 12 months, the Catalog's user interface was accessed by ∼90000 unique users who viewed >1 million pages. We have improved data access with the release of a new RESTful API to support high-throughput programmatic access, an improved web interface and a new summary statistics database. Summary statistics provision is supported by a new format proposed as a community standard for summary statistics data representation. This format was derived from our experience in standardizing heterogeneous submissions, mapping formats and in harmonizing content. Availability: https://www.ebi.ac.uk/gwas/.

## INTRODUCTION

**Figure 1.**
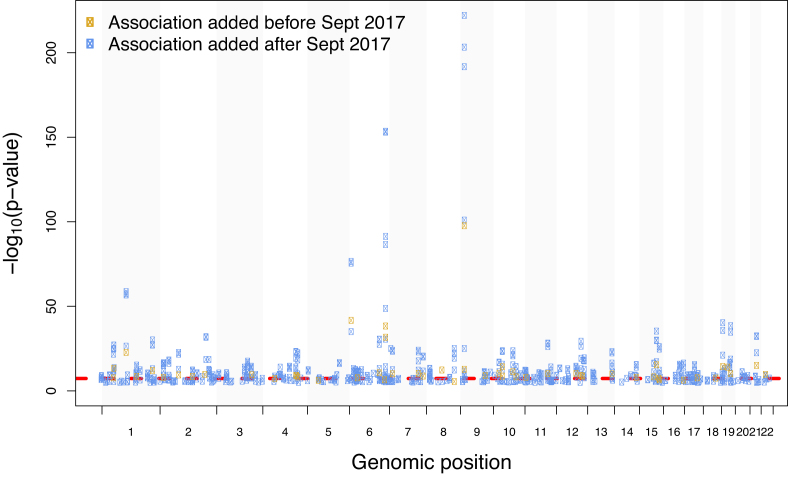
GWAS Catalog associations for coronary artery disease plotted across all chromosomes. Associations added after 2017 represented in blue, previous associations in orange. The dashed red line indicates genome-wide significance at *P*-value = 5 × 10^−8^.

For over a decade, genome-wide association studies (GWAS) have contributed to the identification of reproducible genomic regions associated with an impressive number of common traits, including breast cancer (1,2), ovarian cancer (3), coronary artery disease (4), type 2 diabetes (5), osteoarthritis (6) and systemic lupus erythematosus (7). Further, Nelson *et al.* estimate that the inclusion of genetic associations in the drug discovery process could double the success rate of targets in clinical development (8). The GWAS landscape has evolved over the last ten years with an increase in publications employing complex methodologies, such as trait pleiotropy (9,10), interaction studies (11,12), Mendelian randomization (13) and large meta-analysis (14,15) and this has led to the discovery of new loci. Figure 1 provides an example for coronary artery disease before and after 2017. Many of these new methods require re-analysis of summary statistics results (SS) from GWAS. Full *P*-value SS are defined as the aggregate *P*-values and association data for every variant analysed in an independent GWAS.

The power of recent GWAS has increased through the interrogation of larger sample sizes. For example, UK Biobank (http://www.ukbiobank.ac.uk/) has recruited 500 000 individuals for genotyping, with thousands of phenotypes measured, and the data contributing to 100 GWAS Catalog studies in the last 12 months alone.

The NHGRI-EBI GWAS Catalog (www.ebi.ac.uk/gwas; (16)) is a publicly available resource of published human GWAS. Manual curation of each publication by expert scientists ensures that the Catalog provides accurate and structured metadata for publication, study design, sample and trait information and the most significant published results. Data extracted from publications is now augmented by full *P*-value SS, where available, through the publication or submitted by investigators. The Catalog's data have many applications, for example, to identify causal variants (17), understand disease mechanisms, analyse expression quantitative trait loci (eQTL) (18,19) and establish targets for novel therapies (8,20). In the last year the GWAS Catalog Graphical User Interface (GUI) has been accessed by approximately 90000 unique users and >1 million pages have been viewed, a 40% increase on the previous year. The full Catalog data have also been downloaded 23637 times in the last year with an additional 1281578 downloads of search results and 14653 of summary statistics.

2018 marks the 10th anniversary of the GWAS Catalog. The Catalog has grown 40-fold since the first version which contained 139 studies (21) to 5687 studies and 71673 variant-trait associations from 3567 publications. The growth in the GWAS Catalog scientific content is displayed on the iconic GWAS Catalog diagram, now available as an animation showing the increasing knowledge of traits and their associated variants over time (http://www.ebi.ac.uk/gwas/video/gwas_catalog_2018_anniversary.mp4).

The Catalog's scope has recently been extended in collaboration with Open Targets to serve data from targeted arrays - MetaboChip, ImmunoChip and exome arrays, with the aim of increasing the number of causal variants that focus on immunologic, metabolic and oncologic phenotypes assayed using these technologies. So far, newly curated data on these platforms include 108 targeted studies published between 2008–2018 and 1000 unique SNP-trait variants (manuscript in preparation).

Traits in the Catalog are represented using the Experimental Factor Ontology (EFO, (22)). Supporting semantic tools, the Ontology Lookup Service (23) and Zooma are used to map new traits and provide a consistent and computable representation of traits for users to enhance queries and visualization. Use of EFO also allows the GWAS Catalog data to be interoperable with other datasets as trait annotation is expressed using a formal ontology. Many users map the Catalog's traits to different ontologies. The Ontology Cross Reference Service (OxO) offers mappings to other ontologies, including clinical terminologies such as Snomed-CT (http://browser.ihtsdotools.org/) and the NCI Thesaurus (24) enabling semantic integration of the Catalog's data.

Community engagement has identified two major user groups for the Catalog: those accessing the interface, and those downloading whole, or partial datasets for standalone analyses, or integration into other resources. Therefore, to extend functionality for both user groups we have (i) enhanced search functionality and visualization, (ii) deployed a RESTful Application Program Interface (API), which reduces dependencies on the increasingly complex GWAS Catalog spreadsheet download format, (iii) delivered a new SS database and integrated this with the Catalog's interface and (iv) proposed a candidate format for GWAS SS for community use, and harmonization processes supporting this format (see supplementary materials) with the intention of simplifying data acquisition from the Catalog.

## SUMMARY STATISTICS IN THE GWAS CATALOG

Full *P*-value summary statistics have been available for selected studies from project specific websites for some time, for example, the Psychiatric Genetics Consortium has offered downloads for published studies since 2013 (https://www.med.unc.edu/pgc/results-and-downloads). More recently, funders such as NIH have assessed the risks associated with sharing aggregate genomic information and this has driven current community practice of sharing aggregate SS for GWAS in a project-centric model. This presents challenges for users who must first find, then harmonize, organize and manage the data related to individual studies from multiple sources. This prompted the Catalog to respond to changing community practice and the Catalog now accepts submissions of SS in support of eligible GWAS Catalog papers and has developed new infrastructure to support user access to these data.

**Figure 2.**
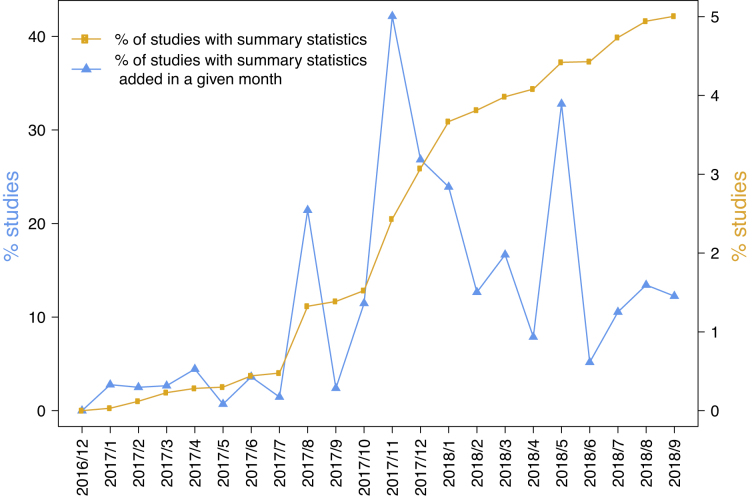
The increasing proportion of studies with available SS in the GWAS Catalog and newly added studies in each month from December 2016, when these were first available.

The SS files are made available through a new Catalog page and each SS dataset is linked to the curated data and metadata from the publication through a unique GWAS study accession number (for example, ftp://ftp.ebi.ac.uk/pub/databases/gwas/summary_statistics/BarbanN_27798627_GCST006045). We have re-curated a set of older publications where the existing data in the GWAS Catalog did not match the required study structure. For example, in a GWAS on human reproductive behaviour, SS were provided organized by sex of the participants (25), whereas the GWAS Catalog studies contained data from both sexes. The existing curated Catalog entries were therefore updated to properly represent the summary statistics. We therefore encourage authors to deposit their SS data in advance of the curation of a related paper to avoid restructuring previously curated data. We have designed a simple SS deposition process using FTP, and in future we will deliver a deposition tool to simplify the process of deposition and link it to curation activities, with the aim of integrating these processes to provide access to richer data as quickly as possible. As of September 2018, 284 SS datasets from 116 publications, representing over 6 × 10^9^ individual variant-trait statistics are available from the Catalog. We encourage authors of all studies in the Catalog to submit their SS and have campaigned for deposition via Twitter (@GWASCatalog). Sharing of SS is also promoted at conferences and we engage with consortia carrying out large-scale genotyping/phenotyping projects. For example, we have worked with representatives from the MAGIC Consortium (www.magicinvestigators.org) and Immunobase (https://www.immunobase.org/) to make available SS from their GWAS studies (26,27). We observe a positive trend within the genetics community in sharing SS; over the last 12 months the proportion of studies with SS in the GWAS Catalog has increased from 1.4% to 5% (Figure 2).

SS, as provided by authors, are available via FTP from the GWAS Catalog. However, the files provided are highly heterogenous with respect to format and content. Organization of individual SS by study does not enable users to query across multiple studies, for example, to retrieve all *P*-values from a given genomic region associated with a particular trait is a common query which is not supported by this organizational model. To address the heterogenous data formatting and content, we analysed the variety of SS files provided to the Catalog. Files were commonly provided as a tab- or comma-delimited format, but column labels were poorly standardized. For example, ‘Chr’, ‘CHR’, ‘Chromosome’ columns were all used, whereas in other case the ‘LOC’ column would contain both the chromosome and base pair location separated by a colon. Sometimes variants were reported by rsID, sometimes by chromosome and base pair location referencing different genome builds, and sometimes by a combination of both. Therefore, we propose a standard set of fields and a standard format, and we have developed a harmonization and QC process in collaboration with Open Targets (28) (see supplementary materials). Our standard format contains a minimal set of requirements that are included in the outputs from the most common GWAS analysis programs (such as PLINK, (29) and additional optional columns.

The size of SS files and the types of queries users need to perform present significant challenges for query performance requiring adjustments to the Catalog informatics infrastructure. We therefore identified a representative set of user queries; for example, retrieving *P*-values and associated fields, *P*-value plus the effect allele frequency, or beta-coefficient and standard error for combinations of variant, trait and study. The existing GWAS Catalog infrastructure uses a relational database for storage and when tested, did not scale to support the necessary range of queries over billions of data points. We evaluated the performance of several alternatives, including a relational database with a simplified GWAS schema to optimize performance, Cassandra and MongoDB. We found that the optimum performance and query times could be achieved using a HDF5 data library, and that queries over this data library scale to support anticipated data volumes over at least the next five years.

A GWAS Catalog summary statistics datastore, based on the HDF5 library format developed in collaboration with Open Targets, is now available for computational access of SS data (see data availability section). The API is developed in Python using the Flask framework and the h5py library, backed by a series of HDF5 files. SS data from the Catalog are processed via a pipeline that implements a harmonization and QC process (see supplementary material), data are then loaded into the datastore, where they are indexed by study, trait, variant and base pair location. This provides rapid access to SS data when querying by one of these dimensions, for example for fine mapping of variants. The GWAS Catalog eligibility criteria have been updated to include SS, and in future all Catalog entries with eligible summary statistics will be loaded into the datastore.

## NEW SEARCH INTERFACE AND API

The Catalog's user groups typically search and visualize data via the GUI, or download some or all of the Catalog's data. Improved search and display functionality are often requested by GUI users, as is access to granular information with genomic context for variants of interest. We have therefore redesigned the query interface and deployed new trait, variant and study pages, integrated access to SS, and improved the specificity of searches. The GUI and API use common core organizing concepts (publication, study, variant and trait) and are based on the same architecture to maintain a common experience for users.

**Figure 3.**
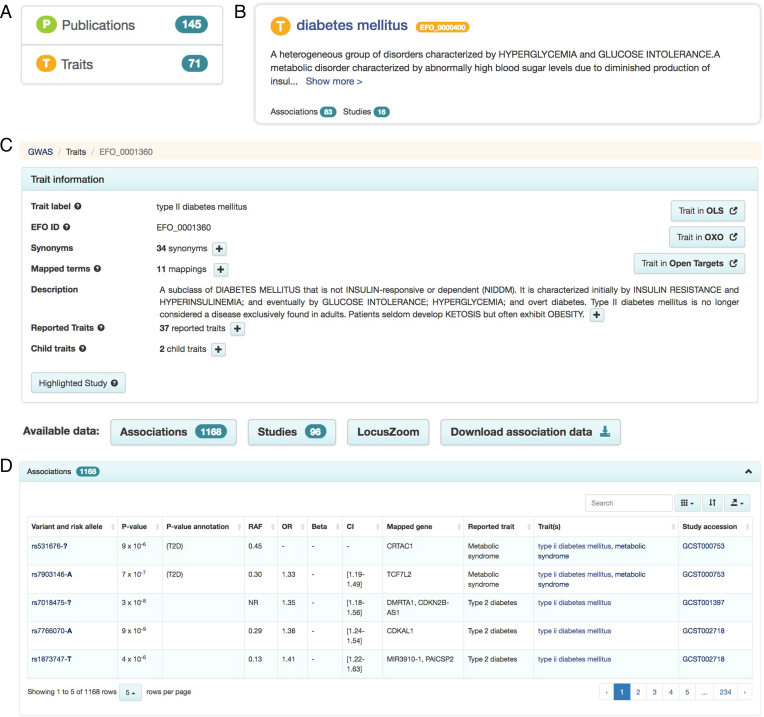
Illustrative query results for diabetes mellitus showing: facets for publications and traits linked to the query term (**A**), a summary of the trait context and ontology definition (**B**), complete trait information with navigation to studies, associations, LocusZoom and data download link (**C**), tabulated variants associated with the query (**D**).

The previous search interface returned all data matching the search term thereby providing a comprehensive but non-specific search. As the data volume has grown this needed to be improved. For example, for a ‘diabetes’ search, users are typically looking for traits matching ‘diabetes’, however, this search also retrieved records containing publication data. Feedback from users indicated this was sometimes confusing. The new search interface was designed after analyzing query patterns from our logs, collating feedback from users during training sessions and combining rapid prototyping with user testing. The new search interface is designed to provide more intuitive access to the most relevant results, according to expected querying behaviour. The improved GUI retrieves ‘diabetes’ as a list of traits containing the term ‘diabetes’, e.g. ‘gestational diabetes’, ‘diabetes mellitus’ and ‘type ii diabetes mellitus’ and facets now show the search result context-trait and publication in this case (Figure 3A). New trait, variant, publication and study pages allow the user to access structured information providing detail and context that were previously unavailable. For example, users could select the trait “diabetes mellitus” (Figure 3B) and access Catalog data for this trait (Figure 3C). To allow users to determine immediately if search results are relevant, contextual summary information is provided in the search results. For example, a query for rs7329174 provides location, cytogenetic region, minor allele, consequence and mapped gene(s) for the variant of interest. The new user interface also supports intuitive navigation between publication, study, variant and trait pages, for example by clicking study accessions in the association table (Figure 3D).

The Catalog has been available as a tab delimited download for 10 years and this requires users to parse and iterate over each biweekly data release for integration of analysis. We have therefore deployed a new RESTful API (www.ebi.ac.uk/gwas/rest/api) that provides programmatic access to the GWAS Catalog data (excluding SS, which are accessible from a dedicated API). Requests are submitted as HTTP and returned in JSON to support additional query granularity requested by programmatic users, for example, ancestry information and author's ORCIDs. Example scripts are available illustrating common queries, for example, retrieval of associations for a trait or variant (www.ebi.ac.uk/gwas/labs/rest/docs/sample-scripts).

## NEW VISUALIZATION TOOLS

**Figure 4.**
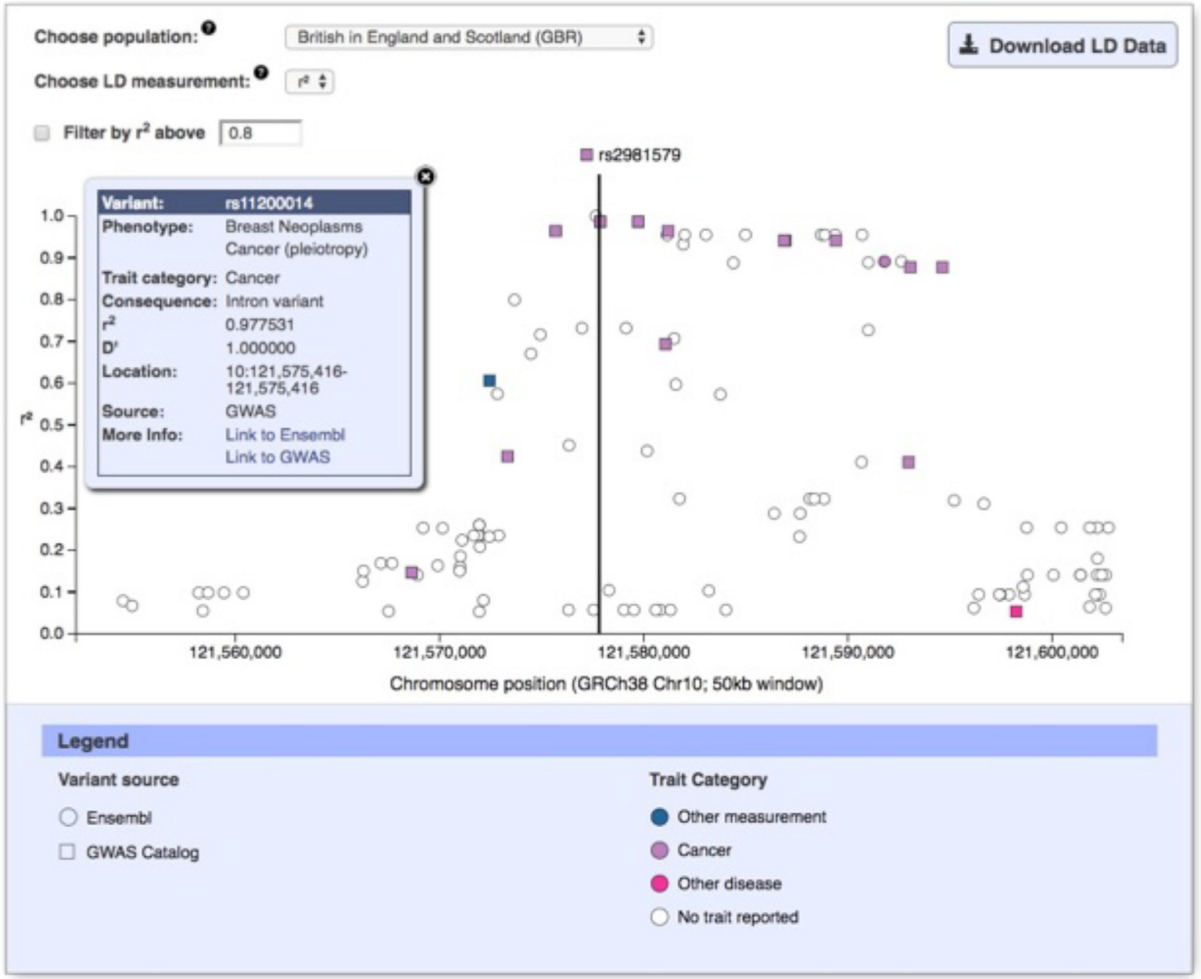
Plot of linkage disequilibrium data for variants within 50 kb of rs2981579.

Variants identified through GWAS are not necessarily causal and typically tag a region of association containing potential causal variant(s). The Catalog data are used to investigate the region of association tagged by a GWAS variant and to prioritize potential causal variants for fine mapping. Therefore, a new interactive query and visualization of the variant's genomic context and linkage disequilibrium in 50 kb window is calculated (as *r*^2^ or D’) for HapMap (30) and 1000 Genomes (31) populations. Variants are coloured using the same scheme as the karyotype diagram allowing easy visualization of traits (Figure 4).

Users often consider GWAS Catalog data from multiple studies when identifying regions of association for use in drug target discovery or disease risk prediction. We have incorporated a LocusZoom plot (32) of all GWAS Catalog associations across the genome by trait to support this. This plot is interactive, allowing users to access data including variant ID, risk allele, study and publication information when hovering over a variant. The interpretation of variants requires users to consider co-located genes and other functional elements. Links to Ensembl (33) and the UCSC genome browser (34) provide genomic context. Finally, we have improved the ease and speed of access to SS, indicating availability through an icon displayed in the search results, study page and study tables. Clicking on the icon from the study page and table provides access to SS files from the FTP site or the API.

## DISCUSSION

The GWAS Catalog has provided access to curated GWAS data for ten years. In the last two years we have significantly extended the Catalog's scope from manual extraction and curation of data from GWAS studies in papers to support changing user needs. We have delivered a new HDF5-based data resource providing access to SS, added targeted arrays and exome studies to the Catalog, and provided a redesigned search interface and API improving access to the Catalog's data. Curation of data from the scientific literature remains a challenging task, both to keep pace with increasingly complex study designs and to deal with the variety of unstructured data in papers. The mean time from journal publication to inclusion in the Catalog is now three months (October 2017–October 2018). We have been successful in engaging the community to deposit SS data, with 11% of studies added to the Catalog in the last year having both curated data and SS. Nevertheless, obstacles remain in the terms and conditions for accessing SS data. We are aware of at least 20 project-specific websites hosting SS which could not be imported into the Catalog due to restrictive licensing and/or terms and conditions. We will therefore engage the funders and policy makers to promote data sharing and clear data licences for SS data to enable us to standardize and share these data. Our SS harmonization process will be offered in the future as a service for users and we will transition to user deposition of first SS, and later GWAS Catalog data, prior to publication via a new submission/curation tool currently being specified. The benefits of this for the user are: data availability immediately at publication and data completeness as SS and GWAS Catalog data can be recruited synchronously. Historically we have accepted only published studies, but this will change to allow the inclusion of UK BioBank data sets, a key resource for the community. Study identifiers will be created for all studies in future, both to allow UK BB data to be accessible from the Catalog and in support of the Findable, Accessible, Interoperable and Re-usable (FAIR) principles (35). We will also encourage the citation of these in the literature in support of the FAIR principles. Inclusion of unpublished data or preprint data raises data quality issues; we will therefore investigate the application of FAIR metrics and GWAS specific QC processes and will tag unpublished or preprint data in the Catalog in future. The GWAS Catalog is a community resource and we will continue to engage the user community, including GWAS data generators, publishers, tools developers and data consumers, to define the meta data, quality criteria and format standards enabling rapid deposition of GWAS data in the Catalog and to ensure the Catalog remains relevant to the scientific aims of our community. We have already surveyed the user community for prioritization of which data to curate and will repeat this in future should bandwidth to curate data change. Our new developments reported here are designed to support genotyping technology extensions in the future, including genome-wide and exome sequencing. We have already performed a pilot study investigating the current volume and data content for sequencing studies. We expect to leverage current standards and add extra columns for sequencing studies to accommodate *P*-values combined for multiple variants. We look forward to community feedback on the new features described here and encourage users to contact our helpdesk via gwas-info@ebi.ac.uk.

## DATA AVAILABILITY

The GWAS Catalog is an open source project and code is available in the project's github repository (https://github.com/EBISPOT/goci). Curated data are available from the query interface (https://www.ebi.ac.uk/gwas/) and download files from https://www.ebi.ac.uk/gwas/downloads. APIs for the summary statistics data (http://www.ebi.ac.uk/gwas/summary-statistics/api/) and the curated data (https://www.ebi.ac.uk/gwas/docs/programmatic-access) provide programmatic access to all the Catalog's data. The Catalog's GUI also provides access to SS https://www.ebi.ac.uk/gwas/downloads/summary-statistics. The Ontology Cross Reference Service (OXO) is available from https://www.ebi.ac.uk/spot/oxo/ and the Zooma annotation-ontology matching tool from https://www.ebi.ac.uk/spot/zooma/.

## Supplementary Material

Supplementary Data
