# Supplementary material for: The NHGRI-EBI GWAS Catalog of published genome-wide association studies, targeted arrays and summary statistics 2019
Source: Nucleic Acids Res. 2018 Nov 16;47(Database issue):D1005–12. doi: 10.1093/nar/gky1120 (PMC6323933; doi:10.1093/nar/gky1120)
Supplement: Supplementary Data [file gky1120_supplemental_files.pdf]

The NHGRI-EBI GWAS Catalog of published genome-wide association studies, targeted arrays and summary statistics 2019

Supplementary Data

SS1. Summary statistics standard format

We propose this tab/CSV delimited format with column labels shown in SS Table 1 as a community standard for summary statistics data files. This is based on analysis of 200 SS files. Each row in in the table below represents a column label. The first four rows contain the minimum amount of data required for the SS to go through the harmonisation pipeline (see SS2). This format was derived from our experience in parsing data files, mapping the formats and harmonising the content. Example data files can be accessed from the SS page (https://www.ebi.ac.uk/gwas/summary-statistics).

Column Label	Status	Definition and Example
‘variant_id’	M	Variant ID e.g. ‘rs7329174’
‘p-value’	M	Reported p-value e.g. ‘2.826e-7’
‘chromosome’	M	Chromosome i.e. 1-25, where 23=X, 24=Y, 25=MT
‘base_pair_location’	M	Location on the current assembly e.g. ‘99534456’
‘effect_allele’	O	Allele associated with the effect e,g. ‘A’
‘other_allele’	O	The reference/wild type/other allele e.g. ‘T’
‘effect_allele_frequency’	O	E.g. ‘0.2449’
‘odds_ratio’	O	E.g. ‘1.27’
‘beta’	O	E.g. ‘-0.0072’
‘ci_upper’	O	Upper 95% confidence interval e.g. ‘1.38’
‘ci_lower’	O	Lower 95% confidence interval e.g. ‘1.17’
‘standard_error’	O	E.g. ‘0.0067’

SS Table 1. Column labels proposed for the SS data file format. Abbreviations: M: mandatory field; O: optional field.

2. Summary statistics harmonisation processes

Harmonisation includes mapping of variants to GRC38, the genome reference build used by the Catalog, identification and removal of unmapped rsIDs and orientation of all the alleles to the forward strand by using the Ensembl Variant Call Format files (https://www.ensembl.org/info/data/ftp/index.html) The harmonisation code is available (https://github.com/EBISPOT/sum-stats-formatter/tree/master/harmonisation) and will be deployed as a service in future. The process is represented as a workflow in Figure SS1.

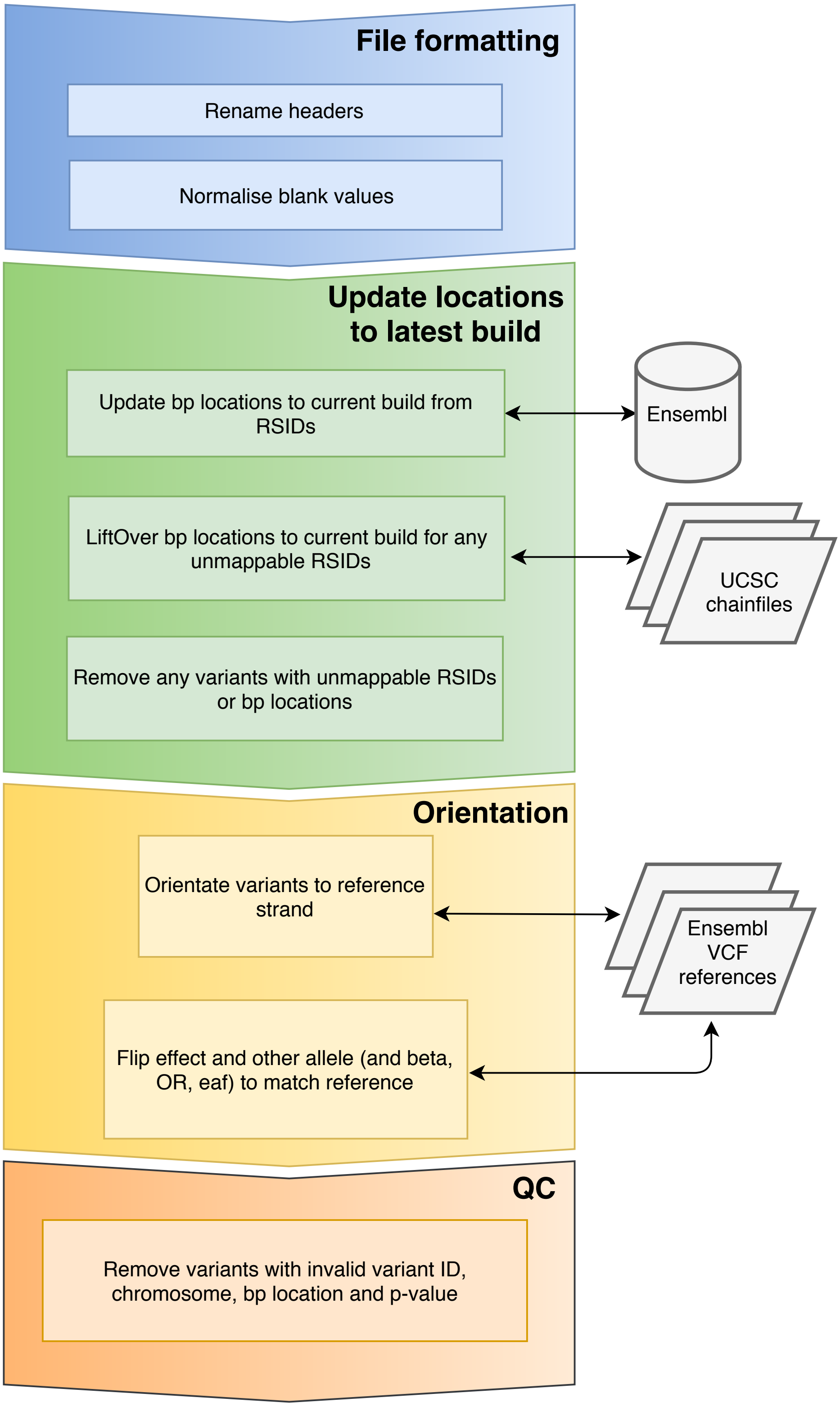

Figure SS1: The summary statistics data harmonisation workflow. Abbreviations: OR: odds ratio; eaf: effect allele frequency.
